# Mineralocorticoid Receptor Antagonism Attenuates Multiple Organ Failure after Renal Ischemia and Reperfusion in Mice

**DOI:** 10.3390/ijms24043413

**Published:** 2023-02-08

**Authors:** Eun Jung Park, Jihyun Je, Theodomir Dusabimana, Seung Pil Yun, Hye Jung Kim, Hwajin Kim, Sang Won Park

**Affiliations:** 1 Department of Pharmacology, Institute of Health Sciences, Gyeongsang National University College of Medicine, Jinju 52727, Republic of Korea; 2 Department of Convergence Medical Sciences, Gyeongsang National University Graduate School, Jinju 52727, Republic of Korea

**Keywords:** renal ischemia reperfusion injury, acute kidney injury, mineralocorticoid receptor, aldosterone, multiple organ failure

## Abstract

Renal ischemia reperfusion (IR) injury is a major cause of acute kidney injury (AKI) that is often complicated by multiple organ failure of the liver and intestine. The mineralocorticoid receptor (MR) is activated in patients with renal failure associated with glomerular and tubular damage. We thus investigated whether canrenoic acid (CA), a mineralocorticoid receptor (MR) antagonist, protects against AKI-induced hepatic and intestinal injury, suggesting the underlying mechanisms. Mice were divided into five groups: sham mice, mice subjected to renal IR, and mice pretreated with canrenoic acid (CA; 1 or 10 mg/kg) 30 min prior to renal IR. At 24 h after renal IR, the levels of plasma creatinine, alanine aminotransferase and aldosterone were measured, and structural changes and inflammatory responses of the kidney, liver, and intestine were analyzed. We found that CA treatment reduced plasma creatinine levels, tubular cell death and oxidative stress induced by renal IR. CA treatment also decreased renal neutrophil infiltration and inflammatory cytokine expression and inhibited the release of high-mobility group box 1 induced by renal IR. Consistently, CA treatment reduced renal IR-induced plasma alanine transaminase, hepatocellular injury and neutrophil infiltration, and inflammatory cytokine expression. CA treatment also decreased small intestinal cell death, neutrophil infiltration and inflammatory cytokine expression induced by renal IR. Taken together, we conclude that MR antagonism by CA treatment protects against multiple organ failure in the liver and intestine after renal IR.

## 1. Introduction

Acute kidney injury (AKI) is defined as an abrupt decrease in renal function. Renal ischemia reperfusion (IR) injury is one of the causes of AKI. AKI is estimated to occur in about 20–200 per million population, 7–18% of hospitalized patients and over 50% of patients in intensive care units [1]. Its progression to end-stage renal disease (ESRD) is a clinically important issue that increases the morbidity and mortality of patients, as well as increases the healthcare-associated costs [2]. Unfortunately, there are currently no known effective therapies for AKI except for renal replacement therapy, including kidney transplantation and dialysis. Importantly, kidney failure due to AKI affects distant organs in the body including lung, liver, heart, intestines and brain, and thus the mortality rates from AKI are high in patients with extrarenal complications, such as pneumonia, acute heart failure, or sepsis [3,4]. Short-term effects of AKI include electrolyte imbalances, metabolic acidosis, and fluid overload, while accumulation of uremic toxins, increased oxidative stress, and impaired immune response contribute to multiple organ dysfunction with long-term effects [5]. However, the pathogenesis of multiple organ dysfunction following AKI is not well understood and novel therapeutic targets or biomarkers may need to be determined [6].

The liver plays a critical role in detoxification, metabolism, and redox balance. The mortality rate in AKI patients with pre-existing liver damage increased from 28 to 58% [7]. AKI upregulates the hepatic levels of oxidative stress, apoptosis, inflammatory cytokines, and immune cell infiltration, which may be related with a significant morbidity [6]. Plasma tumor necrosis factor-α (TNF-α), interleukin-6 (IL-6), and interleukin-17A (IL-17A) levels are increased significantly following AKI. Intestinal Paneth cells synthesize and release IL-17A to initiate intestinal and systemic inflammation [8,9]. In addition, AKI induces intestinal barrier disruption through endothelial and epithelial cell injury, exacerbates systemic inflammation by excreting many uremic toxins derived from intestinal microbiota [3,10]. However, there are no effective therapies to reduce multiple organ dysfunction following AKI, which is essential to lower the high mortality observed in patients with AKI.

Aldosterone is the main mineralocorticoid hormone synthesized in the glomerular zone of the adrenal cortex; aldosterone stimulates renal sodium reabsorption and potassium excretion, playing a major role in the control of blood pressure and extracellular volume homeostasis [11]. The renin–angiotensin–aldosterone system (RAAS) plays a critical role in blood pressure homeostasis; however, it is also involved in the pathogenesis of cardiac and renal diseases by promoting proliferation, inflammation, and fibrosis [12]. The RAAS is activated in AKI, leading to an increase of angiotensin II formation in the kidney that activates proinflammatory cytokines and profibrotic factors; intrarenal RASS is also upregulated in patients with acute tubular necrosis, correlated with the severity of AKI [13,14].

MR antagonism is an efficient treatment to reduce acute and chronic renal injury. Recent studies in humans have reported that mineralocorticoid receptor (MR) blockade by spironolactone (SP) significantly reduced proteinuria in patients with chronic renal failure [15,16]. SP administration reduced tubular injury and oxidative stress in AKI induced by renal IR in rats [17,18]. The podocyte apoptosis induced by aldosterone was attenuated by SP through PI3K/Akt and p38 mitogen-activated protein kinase (MAPK) signaling pathways, indicating a therapeutic potential of MR antagonism in the pathogenesis of glomerulosclerosis [19]. SP also prevents chronic kidney disease (CKD) development in rats following AKI by inhibiting the activation of fibrotic and inflammatory pathways [20]. Thus, MR antagonism is a promising therapeutic approach to treat AKI and prevent its CKD progression. However, the underlying mechanisms of MR antagonism for multiple organ injury after AKI are not clearly shown.

This study investigated whether canrenoic acid (CA), a major biological active metabolite of SP, protects against AKI-induced hepatic and intestinal injury and the underlying mechanisms. We found that MR antagonism attenuated the renal IR-induced systemic inflammation, strongly suggesting the benefit from MR antagonism in treating AKI patients.

## 2. Results

### 2.1. MR Antagonist Decreases Tubular Cell Death and Oxidative Damage Induced by Renal IR

**Figure 1:**
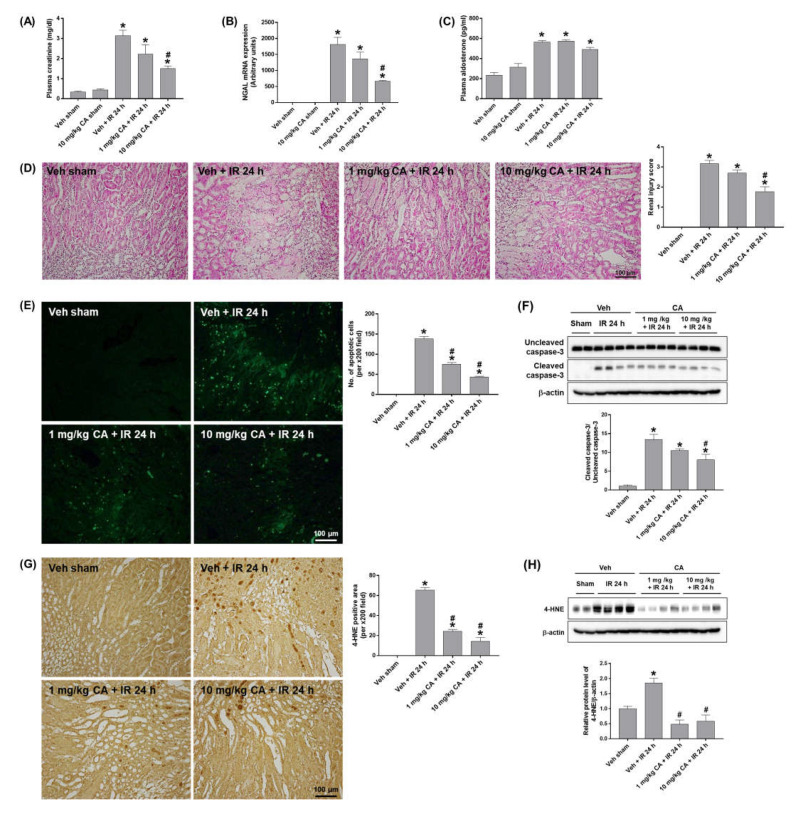
Canrenoic acid (CA) reduces plasma creatinine, tubular cell death, and oxidative stress in renal ischemia-reperfusion (IR) injury. C57BL/6 mice were subjected to 30 min of bilateral renal ischemia. CA (1 or 10 mg/kg) was injected intravenously 30 min prior to ischemia and sacrificed 24 h after reperfusion. Plasma creatinine levels (**A**), renal mRNA expression of NGAL (**B**), and plasma aldosterone levels (**C**). Representative images of H&E staining (**D**) and TUNEL staining (**E**) of kidney sections. The apoptotic cells were counted per ×200 field. Western blots showing the bands of cleaved and uncleaved caspase-3 in kidney tissue lysates; relative protein levels were normalized to levels of β-actin (**F**). Representative images of 4-HNE immunohistochemical staining of kidney sections; the positive cells were counted per ×200 field (**G**). Western blots showing the bands of 4-HNE in kidney tissue lysates; relative protein levels were normalized to levels of β-actin (**H**). The values are expressed as the means ± SEM. * *p* < 0.05 compared to sham group. # *p* < 0.05 compared to IR group.

To investigate the effect of MR antagonism on renal IR injury, mice were treated with canrenoic acid (CA), an MR antagonist prior to renal IR. CA treatment (10 mg/kg) significantly reduced plasma creatinine levels in mice subjected to renal IR (IR mice) but did not induce any renal dysfunction in mice treated CA alone compared to sham mice (Figure 1A). The expression of neutrophil gelatinase-associated lipocalin (NGAL), a biomarker of renal injury, was significantly reduced in IR mice pretreated with CA (10 mg/kg) (Figure 1B). Plasma aldosterone levels were increased in IR mice but not upregulated further by CA treatment (Figure 1C). MR antagonist blockade has previously been shown to upregulate plasma aldosterone levels in patients [21] (Figure 1C). Histological analysis by H&E staining revealed that IR mice exhibited tubular necrosis and proteinaceous casts with increased congestion; however, CA treatment reduced these tubular injuries (Figure 1D). To determine the effect of MR antagonism on the IR-induced renal apoptosis, we performed TUNEL staining and Western blot analysis. IR mice showed a significant increase in tubular apoptotic cells and the levels of cleaved caspase-3, which were reduced by CA treatment (Figure 1E,F). In addition, IR-induced oxidative stress was reduced by CA treatment as shown by a marked reduction in tubular 4-hydroxy-2-nonenal (4-HNE) expression in IR mice pretreated by CA (Figure 1G,H). The results indicate that MR antagonism attenuates renal injury, tubular apoptosis, and oxidative stress in renal IR.

### 2.2. MR Antagonist Reduces Renal Inflammation in Renal IR Injury

**Figure 2:**
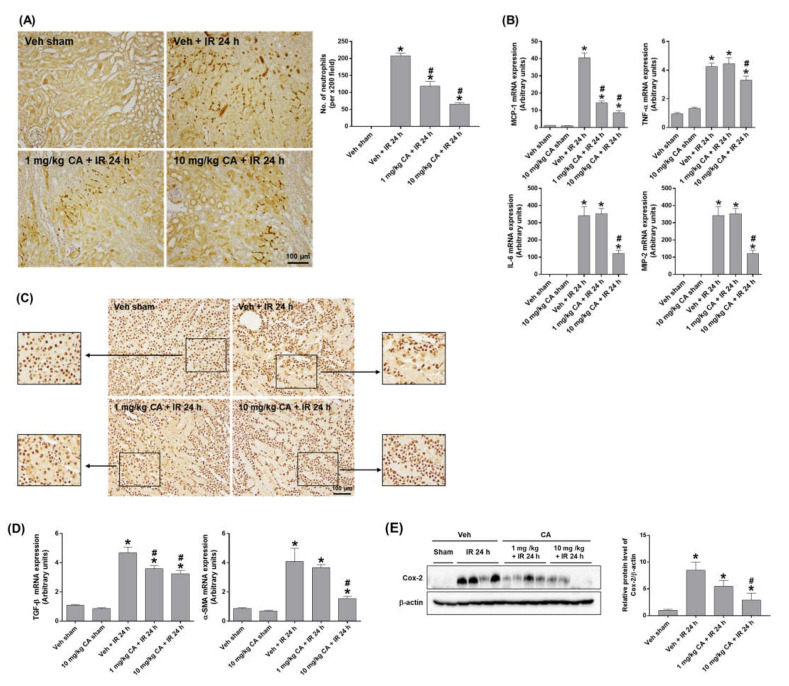
CA reduces neutrophil infiltration, inflammatory cytokines, and HMGB1 release in the kidney of IR mice. Representative images of polymorphonuclear leukocytes (PMN) staining of kidney sections; the positive cells were counted per ×200 field (**A**). Renal mRNA expression of pro-inflammatory genes, MCP-1, TNFα, IL-6, and MIP-2; relative mRNA levels were normalized to levels of GAPDH (**B**). Representative images of HMGB1 immunohistochemical staining of kidney sections (**C**). Renal mRNA expression of profibrotic genes, TGF-β, and α-SMA; relative mRNA levels were normalized to levels of GAPDH (**D**). Western blots showing the bands of COX-2 in kidney tissue lysates; relative protein levels were normalized to levels of β-actin (**E**). The values are expressed as the means ± SEM. * *p* < 0.05 compared to sham group. # *p* < 0.05 compared to IR group.

To determine the effect of MR antagonism on IR-induced renal inflammation, we performed polymorphonuclear leukocyte (PMN) staining. IR mice showed a significant neutrophil infiltration, which was attenuated by CA treatment (Figure 2A). Consistently, upregulation of IR-induced monocyte chemoattractant protein-1 (MCP-1), tumor necrosis factor-α (TNF-α), interleukin-6 (IL-6), and macrophage inflammatory protein-2 (MIP-2) was reduced by CA treatment (Figure 2B). In addition, high-mobility group box 1 (HMGB1) released by activated immune cells was reduced by CA treatment in IR mice. Representative images of immunohistochemical staining of HMGB1 in the kidney showed that the cytoplasmic release of HMGB1 was decreased in IR mice pretreated with CA (Figure 2C), indicating a reduction of extracellular HMGB1 release by CA. The result suggests that MR antagonism attenuated the HMGB1-mediated inflammatory response in IR mice. In ischemic kidneys, MR activation upregulates NADPH oxidase followed by ROS increase, which contributes to endothelial dysfunction and vascular remodeling and stimulates proinflammatory and profibrotic responses [22,23]. As expected, the expression of profibrotic genes, transforming growth factor-β (TGF-β), and α-smooth muscle actin (α-SMA) was significantly increased in IR mice, where CA treatment reduced the expression of TGF-β and α-SMA (Figure 2D). Consistently, the expression of cyclooxygenase-2 (COX-2) was reduced by CA treatment in IR mice (Figure 2E). The results indicate that MR antagonism attenuates tubular inflammation and reduces profibrogenic and oxidative vascular responses induced by renal IR.

### 2.3. MR Antagonist Reduces Hepatocellular Damage and Inflammation in Renal IR Injury

**Figure 3:**
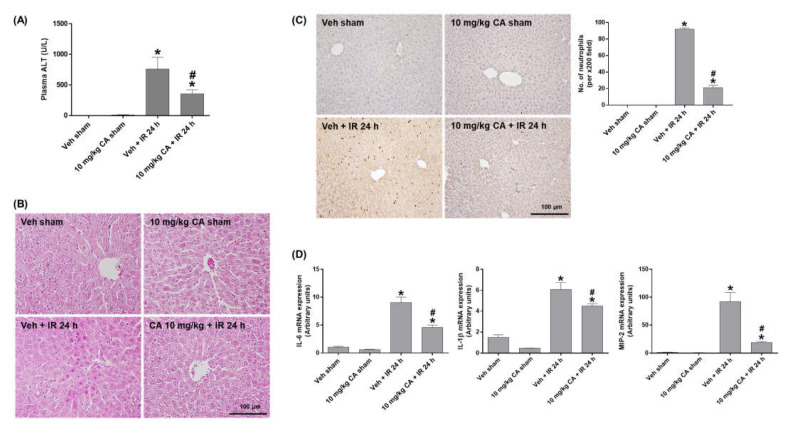
CA reduces hepatocellular damage, neutrophil infiltration, and inflammatory cytokines in the liver of IR mice. Plasma ALT levels (**A**). Representative images of H&E staining (**B**) and PMN staining (**C**) of liver sections; the positive cells were counted per ×200 field. Hepatic mRNA expression of proinflammatory genes, IL-6, IL-1β, and MIP-2; relative mRNA levels were normalized to levels of GAPDH (**D**). The values are expressed as the means ± SEM. * *p* < 0.05 compared to sham group. # *p* < 0.05 compared to IR group.

To investigate the effect of MR antagonism on IR-induced liver injury, we measured plasma ALT levels and performed H&E staining. The ALT levels were significantly increased in IR mice and decreased by CA treatment (Figure 3A). Consistently, IR mice showed a significant increase in necrotic areas and cytoplasmic vacuolization compared to sham mice. However, CA treatment attenuated this hepatocellular damage (Figure 3B). To determine hepatic inflammation, we performed PMN staining and measured expression of pro-inflammatory cytokines. Renal IR increased neutrophil infiltration in the liver, which was attenuated by CA treatment (Figure 3C). In addition, the hepatic expression levels of IL-6, IL-1β, and MIP-2 was increased in IR mice; however, the levels were reduced by CA treatment (Figure 3D). The results suggest that MR antagonism protects against renal IR-induced hepatocellular damage and inflammation.

### 2.4. MR Antagonist Reduces Intestinal Damage and Inflammation in Renal IR Injury

**Figure 4:**
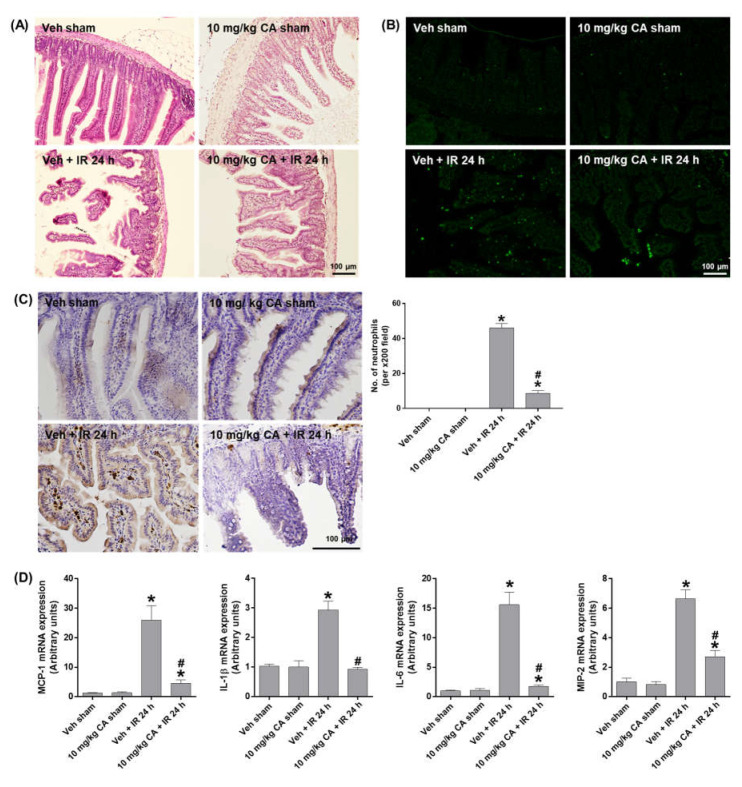
CA reduces small intestinal damage, apoptosis, neutrophil infiltration, and inflammatory cytokines in the small intestine of IR mice. Representative images of H&E staining (**A**), TUNEL staining (**B**), and PMN staining (**C**) of small intestine sections; the number of neutrophils was counted per ×200 field. Intestinal mRNA expression of proinflammatory cytokines, MCP-1, IL-1β, IL-6, and MIP-2; relative mRNA levels were normalized to levels of GAPDH (**D**). The values are expressed as the means ± SEM. * *p* < 0.05 compared to sham group. # *p* < 0.05 compared to IR group.

To investigate whether MR antagonism suppresses epithelial damage and apoptosis in the small intestine after renal IR, we performed H&E and TUNEL staining. Compared to sham mice, IR mice showed a shedding of epithelial cells from the tips of the villi, which was reduced by CA treatment (Figure 4A). The epithelial apoptosis was also reduced by CA treatment in IR mice (Figure 4B). To determine intestinal inflammation, we performed PMN staining and measured the expression of proinflammatory cytokines. IR mice showed a prominent mucosal and mucodermal neutrophil infiltration, which was reduced by CA treatment (Figure 4C). Consistently, IR-induced expression of inflammatory cytokines, MCP-1, IL-1β, IL-6, and MIP-2 was reduced by CA treatment (Figure 4D). The results suggest that MR antagonism attenuates the intestinal tissue injury by reducing epithelial apoptosis and inflammation in renal IR injury.

## 3. Discussion

The present study demonstrated that MR antagonism protects against multiple organ injury induced by renal IR. First, the MR antagonist CA decreased plasma creatinine and NGAL expression, tubular apoptosis, and oxidative stress in IR mice. CA also reduced neutrophil infiltration, expression of pro-inflammatory cytokines, and HMGB1 release in the kidney. Second, the effect of CA in the liver and intestines was determined for remote organ injury after renal IR. CA significantly decreased the hepatocellular damage and inflammation as well as intestinal epithelial damage, apoptosis, and inflammation. In this study, we suggest that MR antagonism may be a potential therapeutic strategy to attenuate renal IR-induced multiple organ injury.

AKI contributes to the development of remote organ failure in the liver, intestine, brain, lung, and heart, which is associated with increased mortality rates in complicated patients [3,4]. The renal IR injury increases tubular cell death and neutrophil infiltration, resulting the release of cytotoxic damage-associated molecular patterns (DAMPs) and proinflammatory cytokines, which enter the circulation and contribute to multiple organ injury [24]. Patients with AKI have significantly elevated plasma cytokine levels, developing a systemic inflammatory response syndrome, which predict high mortality rates [25]. Interestingly, the levels of hepatic TNFα, antioxidant enzymes, and oxidant products are altered 1 h after renal IR in mice, indicating immediate effects on remote organs [26]. Previous studies showed that IL-17A has an important role in renal and hepatic injury after AKI since IL-17A KO mice attenuates organ injury after renal IR [27]. Renal IR- or bilateral nephrectomy-induced AKI increases IL-17A production in intestinal Paneth cells and elevates IL-17A in systemic circulation and delivery to the liver causing hepatic inflammation, necrosis, and apoptosis [9]. In addition, HMGB1 released extracellularly upon renal IR acts as an endogenous ligand for Toll-like receptor 2 and 4 [28], potentially contributing to systemic inflammation and remote organ injury.

In this study, the renal HMGB1 release was increased, and neutrophil infiltration and proinflammatory cytokines were stimulated in the liver and small intestines after renal IR. MR antagonism attenuated the hepatic and intestinal injury by reducing the systemic inflammation promoted by hepatic and intestinal cytokines and renal HMGB1 release, although the detailed molecular mechanisms need to be further determined.

Aldosterone is the final effector of the RAAS, and the increased aldosterone contributes to cardiorenal damage, thus RAAS blockade has been considered to improve cardiovascular disease, hypertension, and diabetic CKD by controlling blood pressure and reducing vascular injury [29]. However, RAAS blockade in patients with perioperative AKI is controversial due to impaired autoregulation of glomerular and arteriolar pressure [30]. A previous study [17] showed that endothelial cell injury and inflammatory responses play a critical role in AKI, suggesting that aldosterone-mediated vasoconstriction may represent an important pathophysiological mechanism of renal IR. Consistently, SP administration prior to renal IR prevents ischemic renal injury due to reduced renal blow flow and creatinine clearance [17]. During a subsequent reperfusion [17] and the development of CKD [20,31], SP treatment reduces tubular apoptosis, oxidative stress, and expression of proinflammatory and fibrotic factors. In this study, we observed that IR-induced renal injury was associated with increased plasma aldosterone levels, supporting a pathological role of aldosterone. We showed that CA treatment reduced liver and intestinal injury and inflammatory responses, suggesting that MR antagonism reduces systemic inflammation [8,9]. Since this study did not investigate the effect of CA on AKI-induced intestinal barrier dysfunction, endothelial and epithelial vascular injuries, and intestinal-derived uremic toxin accumulation, identifying the related signaling molecules is essential for lowering the high mortality observed in patients with AKI.

## 4. Materials and Methods

### 4.1. Experimental Animals

Male C57BL/6 mice (9 weeks old) were purchased from Koatech (Pyeongtaek, Republic of Korea) and maintained in the animal facility at Gyeongsang National University (GNU). All animal experiments were approved by the Institutional Board of Animal Research at GNU and performed in accordance with the National Institutes of Health guidelines for laboratory animal care (GNU-180615-M0028; 16 June 2018). Mice were housed with an alternating 12 h light/dark cycle and provided with water and standard chow ad libitum.

### 4.2. Renal IR Model and Treatment

Mice were divided into five groups: sham mice treated intravenously with vehicle (saline; *n* = 4) or canrenoic acid (CA; Sigma-Aldrich, C7287, Louis, MO, USA) at 10 mg/kg (*n* = 4), and mice subjected to renal IR treated intravenously with vehicle (saline; n = 8) or CA (1 or 10 mg/kg), 30 min prior to renal IR (*n* = 8, each). The mice were intramuscularly anesthetized with zoletil (0.5 mg/kg; Virbac Laboratories, Carros, France) and placed supine on a heating pad under a heat lamp to maintain the body temperature at 37 °C. The kidneys were exposed, right and left renal pedicles were clamped with a microaneurysm clip for 30 min. After ischemia, clips were removed and abdomen was closed by suture. Sham-operated mice were subjected to laparotomy without clamping. The mice were sacrificed 24 h after reperfusion or sham operation. The kidney, liver, and intestine were dissected; the tissues were snap-frozen in liquid nitrogen for storage at −80 °C or fixed in 10% buffered formalin. Blood was collected from an inferior vena cava using a heparinized syringe, centrifuged at 3000× *g* for 20 min, and the supernatants were stored at −80 °C.

### 4.3. Hematoxylin & Eosin (H&E) Staining

After 24 h fixation, the tissues were processed for paraffin embedding and 5 µm-sections were prepared. Sections were stained with H&E (Sigma-Aldrich, St. Louis, MO, USA) by a standard protocol, and images were captured using CKX41 light microscope (Olympus, Tokyo, Japan).

### 4.4. Terminal Deoxynucleotidyl Transferase dUTP Nick End Labeling (TUNEL) Assay

TUNEL analysis was performed by using an In Situ cell death detection kit (Roche Molecular Biochemicals, Mannheim, Germany) according to the manufacturer’s protocol. Images were captured using a Nikon Eclipse Ti-U microscope (Olympus, Tokyo, Japan). The number of positive cells were counted from 5 images of 200× magnification per section from each group (*n* = 3) by using ImageJ software (National institutes of health (NIH), Bethesda, MD, USA).

### 4.5. Immunohistochemistry (IHC)

The fixed tissue sections were deparaffinized, rehydrated, and antigen-retrieved in sodium citrate buffer (10 mM, pH 6.0) for 20 min. The sections were blocked in 10% normal horse serum and incubated with a primary antibody against 4-Hydroxynonenal (4-HNE), HMGB1 (Abcam, Cambridge, UK), or Ly-6B.2 (Bio-Rad, Hercules, CA, USA) overnight at 4 °C. The sections were washed and incubated with a biotinylated secondary antibody (Vector Laboratories, Burlingame, CA, USA) for 1 h at room temperature. Then, the sections were washed again, incubated in avidin-biotin-peroxidase complex (ABC) solution (Vector Laboratories, Burlingame, CA, USA), and then developed using a 3,3′-diaminobenzidine (DAB) peroxidase substrate kit (Vector Laboratories, Burlingame, CA, USA). The sections were dehydrated and mounted using Permount (Sigma-Aldrich, Louis, MO, USA). Images were captured using CKX41 light microscope (Olympus, Tokyo, Japan). The number of positive cells were counted from 5 images of 200× magnification per section from each group (*n* = 3) by using ImageJ software (NIH, Bethesda, MD, USA).

### 4.6. Biochemical Assays

Plasma creatinine was determined by a direct colorimetric Jaffe method and spectrophotometry (Shimadzu UV-1800 spectrophotometer, Tokyo, Japan). Plasma aldosterone was determined using an ELISA kit (Abcam, Cambridge, UK) according to the manufacturer’s instruction.

### 4.7. Western Blot Analysis

The tissues were homogenized and sonicated in ice-cold RIPA buffer (Thermo Fisher Scientific, Waltham, MA, USA) containing protease inhibitors. After centrifugation, the supernatant was collected and protein concentration was determined using a Pierce^TM^ bicinchoninic acid (BCA) protein assay kit (Thermo Fisher Scientific, Waltham, MA, USA). The protein lysates were electrophoresed on polyacrylamide gels and were transferred to polyvinylidene difluoride membranes. After blocking in 5% skim milk, the membranes were incubated with primary antibodies against caspase-3, cleaved caspase-3, cyclooxygenase-2 (COX-2) (Cell Signaling Technology, Danvers, MA, USA); 4-HNE (Abcam, Cambridge, UK), and β-actin (Sigma-Aldrich, Louis, MO, USA). Then, the membranes were incubated with appropriated horseradish peroxidase-conjugated secondary antibodies (Bio-Rad, Hercules, CA, USA) followed by ECL detection (Bio-Rad, Hercules, CA, USA). Relative protein levels were normalized to those of β-actin and quantified using the ChemiDoc XRS + System (Bio-Rad, Hercules, CA, USA).

### 4.8. Quantitative Real-Time Polymerase Chain Reaction (PCR) Analysis

Total RNA was extracted using TRIzol reagent (Thermo Fisher Scientific, Waltham, MA, USA) and 2 μg RNA was converted into cDNA using oligo (dT) primers by the RevertAid Reverse Transcription System (Thermo Fisher Scientific, Waltham, MA, USA) according to the manufacturer’s protocol. Quantitative PCR was performed on a CFX Connect Real-Time PCR System using iQ SYBR Green Supermix (Bio-Rad, Hercules, CA, USA). Thermal cycling conditions consisted of an initial denaturation step at 95 °C for 3 min followed by 45 cycles of 10 s at 95 °C, 10 s at 60 °C, and 30 s at 72 °C. The experiments were carried out in triplicate for each data point. The relative quantification in gene expression was determined using the 2^−ΔΔCt^ method, where Ct represents the threshold cycle for each gene [32]. The fold changes in gene expression were normalized to GAPDH. The primers used in this study are listed in Table 1.

### 4.9. Statistical Evaluation

Statistical difference was determined using one-way analysis of variance (ANOVA, San Francisco, CA, USA) followed by Bonferroni’s multiple comparisons test. The statistical analyses were performed using the GraphPad Prism Software (GraphPad Software Inc., La Jolla, CA, USA). Data were expressed as the mean ± SEM. * *p* < 0.05 vs. sham group; and # *p* < 0.05 vs. IR group were considered as statistically significant.

## Figures and Tables

**Table 1:** The primer sequences used in this study.

Genes	Forward Primers (5′-3′)	Reverse Primers (5′-3′)
GAPDH	GTGGCAAAGTGGAGATTGTTG	TTGACTGTGCCGTTGAATTTG
IL-1β	TCGCAGCAGCACATCAACAAGAG	GGTGCTCATGTCCTCATCCTGGA
IL-6	CCAATTCATCTTGAAATCAC	GGAATGTCCACAAACTGATA
MCP-1	ACCTTTGAATGTGAAGTTGA	CTACAGAAGTGCTTGAGGTG
MIP-2	AGAGGGTGAGTTGGGAACTA	GCCATCCGACTGCATCTATT
NGAL	CACCACGGACTACAACCAGTTCGC	TCAGTTGTCAATGCATTGGTCGGTG
α-SMA	TCAGGGAGTAATGGTTGGAATG	GGTGATGATGCCGTGTTCTA
TGF-β	CGAAGCGGACTACTATGCTAAA	TCCCGAATGTCTGACGTATTG
TNFα	CATATACCTGGGAGGAGTCT	GAGCAATGACTCCAAAGTAG

## Data Availability

Not applicable.
